# Ion Channels and Oxidative Stress as a Potential Link for the Diagnosis or Treatment of Liver Diseases

**DOI:** 10.1155/2016/3928714

**Published:** 2016-01-05

**Authors:** Ana Ramírez, Alma Yolanda Vázquez-Sánchez, Natalia Carrión-Robalino, Javier Camacho

**Affiliations:** ^1^Department of Pharmacology, Centro de Investigación y de Estudios Avanzados del Instituto Politécnico Nacional, Avenida Instituto Politécnico Nacional 2508, 07360 Mexico City, DF, Mexico; ^2^Departamento de Ciencias de la Vida, Universidad de las Fuerzas Armadas, ESPE, Avenida General Rumiñahui, 171-5-231-B Sangolquí, Ecuador

## Abstract

Oxidative stress results from a disturbed balance between oxidation and antioxidant systems. Reactive oxygen species (ROS) and reactive nitrogen species (RNS) may be either harmful or beneficial to the cells. Ion channels are transmembrane proteins that participate in a large variety of cellular functions and have been implicated in the development of a variety of diseases. A significant amount of the available drugs in the market targets ion channels. These proteins have sulfhydryl groups of cysteine and methionine residues in their structure that can be targeted by ROS and RNS altering channel function including gating and conducting properties, as well as the corresponding signaling pathways associated. The regulation of ion channels by ROS has been suggested to be associated with some pathological conditions including liver diseases. This review focuses on understanding the role and the potential association of ion channels and oxidative stress in liver diseases including fibrosis, alcoholic liver disease, and cancer. The potential association between ion channels and oxidative stress conditions could be used to develop new treatments for major liver diseases.

## 1. Introduction

Reactive oxygen species (ROS) and reactive nitrogen species (RNS) are produced during mitochondrial electron transport or by other enzyme systems comprising several oxidoreductases (such as NADPH oxidase which is critical for the bactericidal action of phagocytes) in all cells types, including hepatocytes [1, 2].

ROS play a dual role, because they can be either harmful or beneficial to the cells. The normal physiological ROS-mediated processes include cellular growth, cell proliferation and regeneration, apoptosis, and microbial killing by phagocytes [3]. The most relevant ROS in the cell physiology are superoxide anion (O_2_^−•^), hydroxyl radical (^•^OH), and hydrogen peroxide (H_2_O_2_) while the more common RNS are nitric oxide (NO) and peroxynitrite (ONOO^−•^).

ROS generation is essential to maintain cellular functions and ensure cell survival [4]; this is achieved through the activation of transcription factors, such as NF-kappa-B and hypoxia-inducible-factor-1*α* (HIF-1*α*). ROS also participate in vascular processes [5] and regulate the activation of the immune system by acting as intermediates for cytokines like tumor necrosis factor (TNF-*α*) and interleukin-1*β* (IL-1*β*) [6, 7]. However, overproduction of ROS and/or RNS, associated with a failure in the antioxidant system (superoxide dismutase, catalase, and glutathione peroxidase activities [8]), causes cellular oxidative stress that promotes DNA, protein, and lipid damage, triggering the development of several diseases [9–11].

Many oxidative stress-related diseases have been reported since the first introduction of this term in 1985 by Sies [12]. Some of the most studied conditions are neurodegenerative diseases (Alzheimer, Parkinson, and others) [13–16], cellular aging [17], cardiovascular diseases (hypercholesterolaemia, heart failure, hypertension, myocardial infarction, ischemia/reperfusion injury, and atherosclerosis) [18–20], and pathologies involving chronic inflammation [21]. However, recent studies have also associated oxidative stress with diabetes mellitus [22], obesity [23], infectious diseases (HCV [24], malaria [25]), epilepsy [26], chronic pain [27, 28], and even preeclampsia [29].

## 2. Oxidative Stress and Ion Channels

Ion channels are multimeric proteins located in the plasma membrane and inner cell compartments forming ion-selective pores that open or close in response to specific stimuli such as membrane potential, ligand-binding, temperature, and mechanical stimuli [30]. ROS and RNS can directly induce posttranslational modification of ion channels leading to oxidation, nitrosylation, and/or nitration of specific amino acid residues (sulfhydryl groups or disulfide linkages involving cysteine residues) or indirectly modulate channel function by affecting the signaling pathways that control gene transcription, trafficking, and turnover [31, 32]. The association of ion channels in oxidative stress-related diseases is reported mostly in cardiovascular [33, 34] and neurodegenerative illnesses [35–38]. Multiple studies have reported the involvement of calcium [39–41], potassium [27, 30, 36, 42, 43], sodium [34], and chloride channels [44, 45] in the development of pathologies where oxidative stress plays a major role (Table 1). Ion channels have been also suggested to be associated with oxidative stress in the liver [32, 46] (Table 2). Next, we will describe some of the most studied plasma membrane and mitochondrial ion channels associated with oxidative stress damage, focusing primarily on their potential participation in liver pathological conditions.

### 2.1. Participation of Mitochondrial Ion Channels in Different Pathologies

Generation of high energy molecules is carried out by the mitochondria electron chain transport; hence a great amount of ROS is generated during this metabolism [47, 48]. Because mitochondria are involved in the defense against ROS and its effects on intracellular redox equilibrium, the study of mitochondria in pathological conditions associated with oxidative stress is fundamental [49, 50].

There is a huge diversity of ion channels in both the outer and inner mitochondrial membrane (OMM, IMM). Some of the ion channels located on the outer and inner membrane include voltage dependent anion channel (VDAC), Ca^2+^ uniporter, permeability transition pore (PTP), calcium-activated potassium channels (K_Ca^2+^_), ATP-sensitive potassium channels (K_ATP_), and inner membrane anion channel (IMAC) [51]. The field of mitochondrial ion channels has recently seen some progress through an integrative approach using genetics, physiology, pharmacology, and cell biology tools, which have helped to elucidate the possible functions of these channels [52]. Some of these ion channels participate in several processes such as apoptosis [53, 54], necrosis, thermogenesis, and volume regulation.

The relevance of mitochondrial ion channel research is observed in numerous studies that have demonstrated its participation in the development of pathological conditions like neurodegenerative [35, 55] and cardiac diseases [51, 56–60]. Even though mitochondrial dysfunction is associated with different liver pathologies, such as alcoholic liver diseases (ALD) [49, 50, 61, 62], metabolic syndromes (insulin resistance) [63], and nonalcoholic fatty liver disease (NAFLD) [64], the participation of mitochondrial ion channels in the onset or development of oxidative stress-related liver diseases has not been fully explored.

Nakagawa et al. [65] reported the presence of mitochondrial ATP-sensitive K^+^ (mitoK_ATP_) channels in rat primary hepatocytes. This study demonstrated that diazoxide (a selective opener of mitoK_ATP_ channels) enhanced liver regeneration by keeping a higher ATP content in the liver tissue. They concluded that diazoxide sustains the hepatocytes mitochondrial energetics promoting liver regeneration after partial hepatectomy, which is characterized by the presence of oxidative stress. Indeed, potassium channel openers acting on mitochondria have proved to reduce cell damage observed in ischemia. For instance, the effect of cromakalim and diazoxide on cardioprotection against ischemia-reperfusion injury has been shown [66]. Another study carried out by Shimizu et al. [67] assessed the effect of diazoxide against brain damage after middle cerebral artery occlusion (MCAO) in male Wistar rats. The neurological score was improved in animals treated with diazoxide in comparison with the control group; the effects of diazoxide were prominent in the cerebral cortex. Accordingly, the protective effect was reversed with pretreatment of 5-hydroxydecanoate, a selective blocker of mitoK_ATP_, proving that selective opening of mitoK_ATP_ channel has neuroprotective effects against ischemia-reperfusion injury in the rat brain.

Conductances reported for mitochondrial K_ATP_ channels of different cell types are lower than those reported for plasma membrane K_ATP_ variants, under the same ionic conditions. Moreover, Szabo and Zoratti [52] mention that small-conductance Ca^2+^-activated K^+^ channels have conductances compatible with the lower values reported for mitoK_ATP_ channels. Pharmacological approaches have their own complications due to the nonspecific targeting exhibited by some mitoK_ATP_ inhibitors (5-hydroxydecanoate) and openers (such as diazoxide and cromakalim) which can also act on plasma membrane K_ATP_ channels, according to some studies. Activators of K_ATP_ and SK_Ca_/IK_Ca_ (small-conductance, SK_Ca_; and intermediate-conductance (IK_Ca_) calcium-activated potassium channels) have structural similarities, suggesting the possibility of a pharmacological crossover [52].

Interestingly, mitochondria associated-endoplasmic reticulum membranes (MAMs), which are important for Ca^2+^, lipid, and metabolite exchange, were investigated by Arruda et al. [63] in the liver. They reported that the reorganization of MAMs in a background of obesity resulted in mitochondrial Ca^2+^ overload, which compromised mitochondrial oxidative capacity and increased oxidative stress. Even though this study did not address Ca^2+^ flux in mitochondria, its results indicate that obesity drives an abnormal increase in MAMs formation, along with an alteration in Ca^2+^ flux from the ER to mitochondria. Transient increase in Ca^2+^ level activates mitochondrial matrix enzymes and stimulates oxidative phosphorylation; thus, a high Ca^2+^ flux to the mitochondria is detrimental, promoting oxidative metabolism and consequently ROS production [68].

Mitochondrial ion channels are also considered oncological targets due to the fact that cancer transformation involves reprogramming of mitochondrial metabolic and apoptotic functions, which are necessary to ensure proliferation of neoplastic cells and promote metastasis [53, 69–73].

## 3. Alcoholic Liver Disease

Alcohol is a psychoactive substance with dependence-producing properties and is considered a causal risk factor for a broad spectrum of diseases and injury conditions including alcohol dependence, liver cirrhosis, and cancer [74]. Emerging evidence suggests that alcohol consumption is also related to the incidence of infectious diseases such as tuberculosis and HIV/AIDS [74]. Chronic alcohol (ethanol) consumption is a well-reviewed risk factor of liver diseases [75–78]. In fact, at least 15% of alcoholic cirrhosis cases end up in hepatocellular carcinoma (HCC), the most common type of liver cancer [79], which has one of the highest mortality rates worldwide [80].

Many investigations strongly suggest that liver damage produced by alcohol is mediated through oxidative stress [76, 81–83]. The multistage process observed in ethanol-induced liver diseases (also called alcoholic induced liver diseases (ALD)) covers a broad spectrum of morphological changes from hepatic steatosis (fatty liver), alcoholic hepatitis, chronic hepatitis, and fibrosis ultimately to cirrhosis, which leads to hepatocarcinoma [79]. Ethanol abuse is directly involved in the generation of ROS and RNS that affect the intracellular redox balance within hepatocytes and other cell types in the liver, such as immune cells (neutrophils), sinusoidal endothelial cells (SECs), Kupffer cells (KCs), and hepatic stellate cells (HSCs). Actually, ethanol exposure impairs the structure and function of mitochondria; thus when ROS production is uncontrolled, several responses that promote immune cell activation are triggered [84].

### 3.1. Ethanol-Induced Oxidative Stress

The liver oxidizes and completely metabolizes alcohol, for which cytosolic and mitochondrial enzymes are required. Ethanol metabolism is carried out through three main pathways involving the following enzymes: alcohol dehydrogenase (ADH) and aldehyde dehydrogenase (ALDH), microsomal ethanol oxidation system (MEOS) via catalysis by cytochrome P450 isoenzymes (2E1, 1A2, and 3A4 isoforms) [85], and catalase. Any type of ethanol metabolism will lead to free radical generation that affects the antioxidant defensive system of the cells [86].

Some of the mechanisms by which ethanol impairs the oxidative balance within hepatic cells are acetaldehyde production by ADH, ethanol-induced hypoxia, mitochondria damage, effects on the immune system [87], induction of CYP2E1, mobilization of iron, and alteration of antioxidant enzymes and chemicals [88]. Ethanol metabolism causes depletion of reduced glutathione (GSH) levels and elevates malondialdehyde (MDA), hydroxyethyl radical (HER), and hydroxynonenal (HNE) protein adducts [86], leading to structural and functional abnormalities in the liver. Moreover, alcohol metabolism* via* CYP2E1 activates stress proteins, promotes endoplasmic reticulum stress, and impairs lysosomal function and autophagy [82]. Additionally, some of the mitochondrial alterations caused by ethanol-induced oxidative stress are DNA damage, ribosomal defects, and inhibition of protein synthesis, which in turn affects the integrity of the electron transport chain (complexes I and II) and the oxidative phosphorylation system that is carried by this organelle [50, 79, 89].

### 3.2. Ion Channels in ALD

The association of ion channels in the mechanism of ethanol-induced oxidative stress to the progression of ALD remains elusive and represents a very interesting field of research. The mitochondrial alterations observed under these conditions include the mitochondrial membrane potential and permeability transition (PT) and changes promoting apoptosis [90]. Alteration of mitochondrial membrane potential has been examined in rat hepatocytes exposed to ethanol using rhodamine 123 (Rh123), an indicator of mitochondrial membrane potential. Acute ethanol administration decreased mitochondrial membrane potential in hepatocytes within 30 min, which indicates that mitochondrial alteration is an early event of ethanol-induced hepatocyte injury. Additionally, the increase in PT is induced by opening of the mitochondrial megachannel also known as permeability transition pore (PTP). PTP is regulated by mitochondrial matrix conditions: electrical membrane potential, thiols, oxidants, pH, and calcium concentration; these are factors disturbed as a consequence of ethanol metabolism [91].

Furthermore, Yan et al. [92] evaluated the effect of ethanol on PTP, mitochondrial membrane potential, and intracellular calcium concentration in cultured hepatocytes. Male Wistar rats were administrated intragastrically with alcohol plus olive oil diet; the control group was given an equal amount of normal saline. Ultramicrostructural changes in mitochondria, PTP opening, mitochondrial membrane potential, mitochondrial mass, and intracellular calcium concentration of isolated hepatocytes were measured. The results showed that the mitochondria of the model group had different shapes and that the PTP was disturbed, causing mitochondria swelling. Moreover, mitochondria transmembrane potential was decreased in comparison with the control group. Intracellular calcium concentration was also increased in the liver cells of the group treated with alcohol. These results indicate that ethanol-induced chondriosome injury could be an important early step in ALD pathogenesis.

The molecular nature of PTP is not completely solved. In the last decade findings made by Bernardi and collaborators [93–95] suggested that reconstituted dimers of the F_0_F_1_ ATP synthase (or complex V) form a channel that exhibits identical properties to those attributed to the mitochondrial megachannel. Indeed, dimers of the ATP synthase treated with Ca^2+^ generate currents indistinguishable from MMC, while monomers lack any channel activity, strongly suggesting that PTP forms from a specific Ca^2+^ dependent conformation of the dimers. Moreover, inducers (thiol oxidants, benzodiazepine (Bz-423)) and inhibitors (Mg^2+^, adenine nucleotides) of PTP channel opening have the same effect on ATP synthase. PTP modulators such as membrane potential and matrix pH also constitute key regulators of the ATP synthase. Open questions remain, and further studies are needed to clarify the effect and mechanism of action of other PTP regulators (e.g., rotenone and quinones) and additional issues concerning the dimer hypothesis. According to these findings, it seems that complex V plays a dual function: ATP synthesis and PTP formation.

PTP participates also in mitochondrial calcium release [96]. Pioneer work established its role regulating mitochondrial Ca^2+^ homeostasis in hepatocytes. Cyclosporin A (CsA) is a potent inhibitor of prooxidant-induced release of Ca^2+^ from isolated mitochondria. Pretreatment of hepatocytes with CsA before exposure to prooxidants (*tert*-butyl hydroperoxide, cumene hydroperoxide, or 3,5-dimethyl-*N*-acetyl-*p*-benzoquinone imine) protected hepatocytes from prooxidant injury. This prevented excessive Ca^2+^ cycling (maintaining mitochondrial Ca^2+^ pool) that leads to alterations of the transmembrane potential and ATP synthesis and consequently compromises mitochondrial functioning and cell survival [97].

Participation of HSCs in ALD has been proposed in various studies [98–101]. HSCs are located in the space of Disse (the liver space between a hepatocyte and a sinusoid) and these cells participate in the process of ECM remodeling (collagen secretion, etc.) after liver injury. L-type voltage-operated Ca^2+^ channels (VOCC) regulate calcium entry into the cytoplasm and subsequently cell contraction, which has been well studied in cardiac and smooth muscle cells. HSCs activated by transforming growth factor-*β*1 (TGF-*β*1) express VOCC [102], suggesting that voltage-operated Ca^2+^ channels could also regulate hepatic microcirculation via cell contraction. Itatsu and collaborators [103] evaluated the effect of ethanol on VOCC in HSCs activation. HSCs are known to proliferate in response to liver injury, changing from a “quiescent phenotype” to the “activated phenotype.” This study showed that VOCC expression in activated HSCs is significantly increased after 14 days of ethanol exposure in comparison with untreated cells. Ethanol increases the secretion of TGF-*β*1 that also induces ROS production and downregulates antioxidant enzymes, participating in fibrogenesis and tumorigenesis [104]; however the precise link between TGF-*β*1, oxidative stress, and VOCC remains elusive.

## 4. Nonalcoholic Fatty Liver Disease

Nonalcoholic fatty liver disease (NAFLD) is the most common liver disease reported in western nations. NAFLD prevalence is 30–45% in Americans. Hispanics have the highest prevalence, followed by Caucasians and African Americans [105]. NAFLD is the accumulation of fat in hepatocytes corresponding to more than 5% of the liver weight in patients and that is not associated with a significant alcohol consumption (conventionally defined as an ethanol intake >20 g/day). The histopathological spectrum includes steatosis (fatty liver) and nonalcoholic steatohepatitis (NASH) which can progress to cirrhosis and finally to hepatocellular carcinoma. NAFLD is associated with the presence of metabolic syndrome. Steatosis is usually associated with a benign prognosis and does not affect overall survival of patients. NASH represents 2–5% of NAFLD cases and is related to increased mortality [106, 107]. The pathophysiology of NASH is complex, involving free fatty acid accumulation (fatty liver), hepatic inflammation (cytokine production), and oxidative stress and lipid peroxidation, as well as hepatocellular damage with or without the presence of fibrosis [108]. Oxidative stress triggers necroinflammation in the liver and contributes to the pathogenesis of NASH. ROS generated during fatty acid metabolism in microsomes, peroxisomes, and mitochondria are the main source of oxidative stress [109].

Mitochondrial dysfunction and oxidative stress play an important role in the pathogenesis of NAFLD, observed in rodent models as well as in patients [110, 111]. Increased serum oxidative markers such as thioredoxin, oxidized LDL, and malondialdehyde have been reported in NASH patients. Lipid accumulation within hepatocytes impairs oxidative capacity of the mitochondria, stimulating peroxisomal, and microsomal pathways of fat oxidation, which results in lipotoxicity. Oxidative stress then promotes apoptosis of hepatic cells via ATP, NAD, and glutathione depletion, as well as by DNA, lipid, and protein damage [112].

A few studies on the participation of ion channels in the pathogenesis of NAFLD or NASH have been reported. Recent investigations reported that cation channels may generate calcium signals during endolysosomal fusion and vesicle trafficking. Two-pore channels (TPCs) are cation-selective intracellular ion channels, expressed mostly in the endosomal system (TPC1) and on late endosomes and lysosomes (TPC2). Activation of TPCs mediates calcium release from lysosomal stores. TPC2 leads to trafficking defects, promoting hepatic cholesterol accumulation, hyperlipoproteinaemia, and finally NASH. Grimm et al. [113] explored the role of TPC2* in vitro* and* in vivo*. Embryonic mouse hepatocytes lacking TPC2 displayed a significant impairment of LDL-cholesterol and EGF/EGF-receptor trafficking which can be attributed to a dysfunction of the endolysosomal degradation pathway. TPC2-deficient mice also presented cholesterol overload and liver damage consistent with NAFLD. These results suggest that TPC2 plays an important role in trafficking in the endolysosomal degradation pathway of lipids and is potentially involved in the homeostatic control of various molecules.

NAFLD and obesity are frequently associated between them and are characterized by the formation of protein inclusions and ubiquitinated proteins. Moreover, it has been suggested that autophagy deregulation during obesity contributes to protein inclusion and progression to fatty liver pathologies, such as steatohepatitis and HCC. To elucidate how lipotoxicity and obesity can affect autophagy, an* in vitro* system in HepG2 cells was established. HepG2 cells cultured with saturated fatty acid exhibited the accumulation of ubiquitinated proteins in soluble inclusion bodies. Increased cytosolic calcium levels in the hepatocytes were observed in an obesity mouse model. The calcium channel blocker verapamil was proven to restore autophagic flux and suppress protein inclusions in both models. Verapamil also reduced hepatic lipid droplet accumulation, insulin resistance, inflammation, and steatohepatitis, which suggest that calcium channel blockers can be used in NAFLD pathologies [114, 115].

## 5. Fibrosis

**Figure 1:**
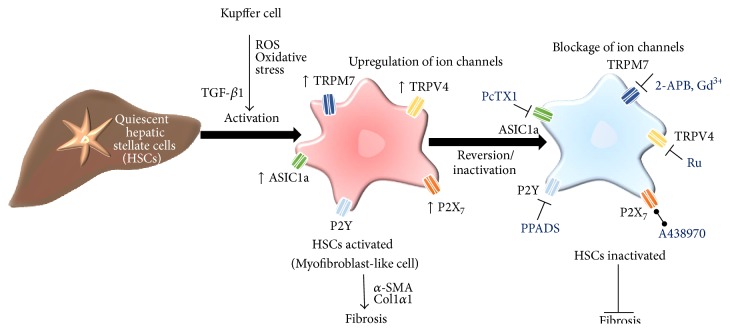
Participation of ion channels in HSCs activation during fibrogenesis. Ion channel upregulation including TRPM7, TRPV4, P2X_7_, and ASIC1a has been reported during the activation of HSCs, which is a major event during fibrogenesis. Blocking these channels with pyridoxal-phosphate-6-azophenyl-2′,4′-disulfonate (PPADS), 2-aminoethoxydiphenyl borate (2-APB), and Gd^3+^; ruthenium red (Ru); PcTX1 or A438970 reduces proliferation of HSCs and production of profibrotic markers (*α*-SMA, Col1*α*1), preventing the progression of fibrosis.

Hepatic fibrosis is characterized by the excessive generation and accumulation of extracellular matrix (ECM) constituents, particularly collagen (types I and III) and fibronectin. Liver fibrosis is a progressive pathology resulting in cirrhosis [116] and ultimately contributes to the development of hepatocellular carcinoma, a malignancy of global importance with very poor prognosis [117]. A fundamental cell event in this process is the activation of HSCs into fibrogenic myofibroblast-like cells which is characterized by the expression of alpha-smooth muscle actin (*α*-SMA) [118]. Following hepatic injury, HSCs become activated by cytokines and ROS released from KCs, causing HSCs to proliferate, synthesize, and secrete ECM components (Figure 1) [119–121]. Liver fibrogenesis is strongly associated with oxidative stress and it may be the liver disease most frequently related with changes in ion channels. Several studies of ion channels and liver fibrosis are next described.

### 5.1. Purinergic Receptors

Purinergic receptors have binding sites for nucleotides like ATP and are divided into ligand-gated (P2X) and G protein-coupled (P2Y) receptors [122, 123]. Diverse roles of the purinergic signals in the liver have been described in recent years. For example, purinergic receptors are involved in the proliferation [124] and glucose secretion [125] of hepatocytes. They are also related to the secretion [126, 127], proliferation [128], and mechanosensation [129] in cholangiocytes. The first evidence relating purinergic receptor and fibrosis showed the presence of functional P2Y2, P2Y4, and P2Y6 receptors in both quiescent and activated HSCs [130]. Dranoff et al. [131] demonstrated that the administration of pyridoxal-phosphate-6-azophenyl-2′,4′-disulfonate (PPADS), a synthetic inhibitor of P2Y receptors, markedly inhibited carbon tetrachloride- (CCl_4_-) induced liver fibrosis in rats [131].

One of the most studied channels described in liver fibrosis is P2X7 [132]. Increased mRNA and protein expression of P2X7 was observed in CCl_4_-induced liver fibrosis in mice compared with vehicle-treated mice. The competitive P2X7 receptor antagonist A438970 significantly attenuated the CCl_4_-induced necrosis, inflammatory infiltration, and cell injury. The antagonist also reduced collagen accumulation and the production of the proinflammatory mediators TNF-*α* and IL-1*β*; in addition it inhibited the activity of NF-*κ*B during inflammation as well as protein expression of profibrotic factors including *α*-SMA and TGF-*β*1 [117]. Das et al. [133] demonstrated that mRNA expressions of *α*-SMA, collagen type 1 alpha 1 (Col1*α*1), and TGF-*β*1 were significantly decreased in P2X7 gene-deleted mice compared with wild type mice, suggesting that P2X7 gene-deleted mice are protected from fibrosis. mRNA expression of P2X receptors in human LX-2, hTERT, and FH11 hepatic stellate cell lines has also been demonstrated [134].

### 5.2. TRPM7 Channels

Transient receptor potential (TRP) channels are a superfamily of cation channels that play critical roles in detecting environmental changes and stimuli. The TRP melastatin-like 7 (TRPM7) channel is selective mainly for Ca^2+^ and is involved in sustaining intracellular Ca^2+^ homeostasis. TRPM7 transcripts are detected in the liver of zebrafish larvae [135] and rat liver [136]. The rat embryonic hepatocyte line RLC-18 expresses a TRPM7-like current suggested to be associated with the proliferation and differentiation of hepatocytes [137]. Likewise, TRPM7 is expressed in HSCs and liver cells from a rat hepatic fibrosis model [138–141]. It is also expressed in rat hepatocytes, the rat hepatoma cells WIF-B cells, and a polarized cell line derived from rat hepatoma-human skin fibroblast cross [139], as well as in H4-IIE cells (rat hepatoma cell line) [140]. Particularly, TRPM7 protein was elevated in fibrotic human liver tissues compared with normal liver tissue and the upregulation of the channel strongly correlated with the increasing levels of *α*-SMA and Col1*α*1 proteins. Additionally, cultured rat HSC-T6 cells treated with TGF-*β*1 showed increased expression of mRNA and protein levels in a time-dependent manner by mechanisms related to the activation of the TGF-*β*1/Smad3 pathway. The elevated channel levels were correlated with the increasing levels of *α*-SMA and Col1*α*1 proteins [141]. Other studies demonstrated that rat primary HSCs displayed increased TRPM7 expression with different stimuli including TGF-*β*1 or platelet-derived growth factor (PDGF-BB), which are some of the main growth factors stimulating the proliferation of cultured-activated HSCs. This cell proliferation was decreased by the treatment with different TRPM7 nonspecific inhibitors such as 2-aminoethoxydiphenyl borate (2-APB) and Gd^3+^. Both inhibitors were able to decrease cell viability in a dose-dependent manner via the activation of the apoptotic pathway [138, 142, 143] and decreased the expression of *α*-SMA and collagen I. Indeed, the treatment with 2-APB and Gd^3+^ inhibited TRPM7 protein expression [143]. It has been also suggested that silencing TRPM7 in the activated HSCs may promote collagen degradation by increasing the levels of hepatic matrix metalloproteinases (MMPs) such as MMP-13 and decreasing the levels of tissue inhibitors of metalloproteinases (TIMPs) like TIMP-1 and TIMP-2 expression [141].

### 5.3. TRPV4

Increased mRNA and protein levels of TRP vanilloid 4 (TRPV4) have been detected in CCl_4_-treated rat livers and in cultured rat HSC-T6 cells. Additionally, the expression of *α*-SMAD and Col1*α*1 were elevated according to the progression of HSC-T6 cell activation and correlated with the levels of TRPV4. The blockage of the channel with ruthenium red (a nonspecific TRPV4 channel blocker) or TRPV4 silencing inhibited the proliferation of TGF-*β*1-treated HSC-T6 cells and decreased profibrotic marker expression. The TRPV4 expression was directly regulated by miR-203 in TGF-*β*1-induced HSCs. Interestingly, increased TRPV4 protein expression was found in the liver tissues from liver fibrosis patients compared to normal liver [144].

### 5.4. Large Conductance Ca^2+^ and Voltage-Activated K^+^ Channels

The large conductance Ca^2+^ and voltage-activated K^+^ (K_Ca_1.1, BK) channels are activated by membrane depolarization and/or elevations in intracellular Ca^2+^ concentration. They are expressed in almost every tissue in the body participating in numerous cellular functions including the regulation of neurotransmitter release and neuronal excitability, relaxation in smooth muscle cells, hormone release in endocrine and exocrine cells, and blood pressure control [145, 146]. Recently attention has been drawn to BK channels as critical targets of oxidative stress which modifies the gating properties of the channel and is associated with numerous diseases [147], mainly those associated with vascular impair, vascular relaxation, and restricted blood flow [148]. Contraction of smooth vascular cells is given by high elevations of intracellular Ca^2+^ which makes the plasma membrane permeable to K^+^ ions by activating BK channels which hyperpolarizes the membrane and causes relaxation [149]. The participation of BK channels has been studied in the modulation of the intrahepatic vascular tone in normal and cirrhotic livers using male Wistar rats exposed to CCL_4_. Cirrhotic livers displayed increased activity of BK channels and blockage of the channel increased the baseline portal perfusion pressure in cirrhotic livers [150]. They also used a vasoconstrictor compound (methoxamine) in combination with a channel opener (NS1619) observing a decrease in the baseline portal perfusion pressure in cirrhotic livers, which indicates the participation of BK channels in the modulation of the intrahepatic vascular tone in cirrhosis. Also, in normal human livers, incubating HSCs with the vasodilator NO (nitric oxide) increases the open probability of BK [151]. ROS have been considered to inhibit vascular BK channels. Tang et al. [152] have found that H_2_O_2_ greatly inhibits BK channels by oxidizing a single cysteine residue (Cys911) near the intracellular Ca^2+^ binding site (Ca^2+^ bowl) in the BK *α* subunit, disrupting the Ca^2+^-dependent activation of the channel. These channels have big amounts of cysteines and methionines in their *α* and *β* subunits [32, 147]; oxidative molecules could cause alterations in these amino acids and therefore impair the channel activity causing important pathophysiological alterations, as it has been seen in vascular relaxation and blood pressure in different diseases.

### 5.5. Chloride Channels

Chloride channels are ubiquitously expressed and are localized in the plasma membrane and intracellular organelles. These channels participate in cell volume regulation, maintain intracellular pH, and are involved in transepithelial transport, cell cycle, and electrical excitability [153]. Nonspecific chloride channel blockers including 4,4′-diisothiocyanatostilbene-2,2′-disulfonic acid disodium salt hydrate (DIDS), 5-nitro-2-(3-phenylpropyl-amino)benzoic acid (NPPB), and indanyloxyacetic acid (IAA-94) prevented the increase of intracellular levels of O_2_^−•^ and inhibited the activation of the HSC human cell line LX-2, induced by the free radical. These findings suggest that the O_2_^−•^ radical may enter through chloride channels producing the HSC activation which is critical to fibrosis development [154]. Other authors, Hawkins et al. [155], have demonstrated that O_2_^−•^ flux across the endothelial cell plasma membrane of immortalized human pulmonary microvascular endothelial cells (HPMVEC) occurs through ClC-3 channels and induces intracellular Ca^2+^ release, which activates mitochondrial O_2_^−•^ production. Also, studies in erythrocytes [156] and amniotic cells [157] have provided evidence for O_2_^−•^ transport through anion channels, which could be effectively blocked by DIDS.

### 5.6. Acid-Sensing Ion Channels

The acid-sensing ion channels (ASICs) are members of the degenerin/epithelial Na^+^ channel superfamily, which are activated by extracellular protons and induce an amiloride-sensitive cation current (Na^+^ > Ca^2+^ > K^+^) [158]. The ASICs family is composed of mammals by four different genes encoding seven isoforms: ASIC1a, ASIC1b, ASIC1b2, ASIC2a, ASIC2b, ASIC3, and ASIC4 [159]. ASIC1a, which is also permeable to Ca^2+^, may play a role in liver fibrosis because the channel mediates the activation of HSCs. ASIC1a is normally expressed in rat liver tissue including primary HSCs, while protein levels were significantly increased in liver fibrosis induced by CCl_4_ where the channel was mainly expressed in activated HSCs. Furthermore, the protein levels of ASIC1a increased in a dose- and time-dependent manner in PDGF-activated HSCs while the blockade of ASIC1a by Psalmotoxin (PcTX1) or the downregulation of the channel by si-RNA reduced the activation of HSCs through the mitogen-activated protein kinases (MAPK) signaling pathway; also ASIC1a silencing inhibited extracellular-signal-regulated kinases 1 and 2 (ERK1/2) and c-Jun N-terminal kinases (JNK) activation [160].

## 6. Cancer

HCC represents 80% of primary liver cancers; the causes leading to HCC include hepatitis B and hepatitis C virus infection (HBV and HCV, resp.) alcoholism, NASH, and aflatoxin B_1_ dietary exposure. These factors produce chronic inflammation with severe oxidative stress leading to fibrosis and then cirrhotic livers; this is a common initial mechanism for HCC [161, 162]. Several ion channels have been studied in HCC. Below we summarize recent studies that associate oxidative stress and ion channels in liver carcinogenesis.

### 6.1. K^+^ Channels

Potassium channels play an important role in various biological processes including cell proliferation, apoptosis, cell volume regulation, and migration and angiogenesis of a variety of carcinoma cells [43]. Different K^+^ channels have been reported to be involved in the pathogenesis of HCC. The intermediate-conductance Ca^2+^ activated K^+^ channel 3.1 (IKCa1, K_Ca_3.1) is overexpressed in HCC tissue [163]. Channel blockade with TRAM-34 inhibited HCC cell proliferation in a time- and dose-dependent manner [163, 164]. TRAM-34 inhibited the activation of NF-*κ*B [164] and activated the MAPK signaling pathway in the SK-Hep1, an invasive liver cell line. The activation of this pathway is linked with the progression of malignant carcinomas [165]. K^+^ channels control the membrane potential; thus overexpression in malignant cells is correlated with the progression of the cell cycle from G1 into the S phase, since a transient hyperpolarization is required in this step of the cell cycle. K_Ca_3.1 could maintain the hyperpolarized membrane potential in cancer liver cells, which promotes Ca^2+^ influx and facilitation of mitogenic activation [166]. Astemizole, a nonspecific inhibitor of K_v_10.1 and K_v_11.1 potassium channels, significantly decreased cell proliferation and increased apoptosis* in vitro* in the liver cancer cell lines HepG2 and HuH7 [167]. In the same study, the authors observed that astemizole clearly prevented HCC development* in vivo*. Another recent finding [168] reported that several potassium channels were overexpressed in the liver only in the presence of the chemical carcinogen diethylnitrosamine; when the carcinogen treatment finished, the channel mRNA levels returned almost to normal values. The authors suggested that some potassium channels may serve as carcinogen exposure indicators and that gene expression of the Abcc3 transporter may serve as a liver tumor marker.

### 6.2. Chloride Channels

In response to oxidative stress in hepatocarcinoma, two types of chloride channels have been studied, the volume-sensitive outwardly rectifying (VSOR) Cl^−^ channel and chloride intracellular channel 1 (CLIC1). VSOR Cl^−^ channels are ubiquitously expressed in various cell types and are involved in cell volume regulation after osmotic swelling (regulatory volume decrease, RVD); but they also participate in cell proliferation and apoptosis [169]. In rat hepatoma cells (HTC), H_2_O_2_ enhances Src mediated PLC*γ*1 phosphorylation, which subsequently increases intracellular Ca^2+^ levels that activate Ca^2+^-sensitive pathways causing VSOR Cl^−^ channel activation [170]; these channels are also activated by H_2_O_2_ in HeLa cells and in both cell lines participate in cell volume regulation and cell proliferation [169]. Thus, activation of VSORs in HTC may provide advantages in the progression of cancer, but further studies are needed to elucidate the potential mechanisms involved. CLIC1 protein has been found to be overexpressed in liver cancer tissues compared to noncancerous liver tissue and significantly correlated with tumor size, metastasis, and poor prognosis [45]. CLIC1 is overexpressed and promotes cell proliferation, migration, and invasion in the mouse hepatocarcinoma ascites cell line Hca-F that has the potential to produce lymphatic metastasis. In accordance, silencing CLIC1 gene expression with shRNA inhibited cell proliferation, induced apoptosis, and decreased migration and invasion [171]. One of the possible mechanisms by which CLIC1 mediates invasion is by regulating maspin (tumor suppressor), matrix metalloproteinases [172], annexin A7, and gelsolin [173].

### 6.3. T-Type Ca^2+^ Channels

Oxidative stress induces Ca^2+^ cytoplasmic increase via calcium influx through plasma membrane channels or calcium release from the endoplasmic reticulum, increasing calcium influx into the mitochondria and nuclei, where different signaling pathways take place in the presence of Ca^2+^ [174]. T-type calcium channels play a role in cell cycle progression in different types of cancer [70, 175, 176]. The expression of the three T-type calcium channel subunits was observed in six HCC cell lines (HuH-1, PLC/PRF5, SMMC7721, SNU182, SNU449, and SNU475) and in the cell line SNU449 T-type channel blockage with mibefradil decreased cell proliferation [177].

### 6.4. P2Y Receptor

Extracellular nucleotides, such as ATP, are released from cells in response to various stimuli, such as shear stress, stretching, hypoxia, inflammation, osmotic swelling, and cell death. In HCC cell lines, the levels of P2Y2 receptor are enhanced compared with human normal hepatocytes; these receptors are involved in ATP-induced [Ca^2+^]_i_ increase. Silencing P2Y2R expression inhibited ATP-induced human HCC cell proliferation and migration, and, in nude mice that were implanted with human HCC cells, blocking P2Y2R inhibited cell growth [178].

### 6.5. Hepatitis C Virus

Hepatitis C virus (HCV) infection is a major health problem worldwide; an estimated 130–170 million people of the world's population are infected with HCV [179]. Most of these individuals (around 80%) will develop chronic liver disease predisposing them to fibrosis and serious clinical outcomes such as cirrhosis and hepatocellular carcinoma [180]. HCV is an enveloped positive-single stranded RNA virus whose genome encodes a single polyprotein that produces mature proteins in the host cell: mature viral structural proteins (Core, E1, E2, and possibly p7) and nonstructural proteins (NS2, NS3, NS4A, NS4B, NS5A, and NS5B) [181]. Chronic HCV infection is associated with elevated levels of ROS and RNS leading to an overall increase of oxidative stress within the liver of patients; this redox perturbation has been recognized as a key player in HCV-associated liver diseases. Ion channels have been involved in the development of the redox state in hepatocytes [182, 183], but their role in hepatitis C virus infection remains largely unstudied. However, some studies have reported changes in ion channels in the presence of HCV.

### 6.6. K^+^ Channels

Voltage-gated potassium channels are essential for several cellular processes participating in proliferation, migration, survival, and apoptosis [184, 185]. In HCV-infected cells, the NS5A viral protein inhibits apoptosis in response to oxidative stress by disrupting the function of the K_v_2.1 channel [24]. In response to oxidative stress, mixed lineage kinase 3 (MLK3) is activated leading to activation of p38-MAPK that phosphorylates K_v_2.1 at a serine residue in the cytoplasmic C terminus (S800), which is then trafficked to the plasma membrane resulting in an outward K^+^ current, which is involved in causing apoptosis. NS5A inhibits MLK3 activation then preventing K_v_2.1 phosphorylation and channel activity in the plasma membrane and avoiding apoptosis [186]. This is advantageous to the infected cells that are able to avoid apoptosis induced by oxidative stress.

### 6.7. Chloride Channels

Activation of chloride channels is required for the life cycle of HCV; blocking chloride channels inhibits HCV genome replication and reduces NS5A expression [187]. This might occur because Cl^−^ channels are responsible for endosome and lysosomal acidification [153], which is necessary to initiate entry fusion of HCV envelope proteins to the membrane and viral genome release [188]. HCV induces ROS production in liver infected cells and activation of chloride channels may take place to maintain the oxidative state in the infected cells. It has been proposed that when ROS are produced by the NAPDH oxidase, the transfer electrons from the intracellular donor NADPH to extracellular oxygen gives rise to an outward flow of negative charges that depolarizes the plasma membrane in leukocytes. To counteract the more negative potential, the activation of chloride channels may take place modifying the membrane potential and allowing an optimal ROS production [44, 189]. Thus, HCV-infected cells may balance the loss of negative charges in the cell by activating chloride channels.

### 6.8. P2X Receptors

P2X receptors regulate proliferation and glucose release in hepatocytes [125, 190]. Transcripts of P2X1, P2X2, P2X3, P2X4, and P2X7 have been observed in rat liver cells and rat hepatocytes [125]. Huh-7 cells transfected with the HCV proteins E1E2 showed increased P2X4 gene expression in comparison with control Huh-7 cells [191]. P2X4 is one of the most responsive subtypes of P2X receptors and may participate in mediating glucose-release via glycogenolysis [192].

## 7. Conclusions

Ion channels play an important role in cellular processes associated with oxidative stress in health and disease. These proteins are very important pharmacological targets for several human pathologies. Therefore, more research on the participation of ion channels in oxidative stress-associated liver diseases should help to find new early-detection tools as well as novel therapeutic strategies.

## Figures and Tables

**Table 1:** Ion channels involved in oxidative stress-related diseases.

Type	Channel	Type of dysregulation	Oxidative stress-related disease	Model	Alteration/pathophysiological effect	Ref.
Na^+^ voltage-gated sodium channels (VGSCs)	Na_v_1.1	Missense mutation	Idiopathic epilepsy	Patients	Increase in sodium influx. Patients show variable seizure types, including absence, myoclonic, tonic-clonic, and partial seizures.	[26, 37, 193–196]
	Na_v_1.2	Missense mutation				
	Na_v_1.5	Punctual mutation (SNP)	Coronary microvascular dysfunction and ischemic heart disease (IHD)	Patients/population study	Polymorphism rs1805124_GG associated with a higher risk to develop IDH.	[34, 193, 195]
	Na_v_1.6	Large persistent sodium current	Neurodegenerative diseases	Mouse model	The large persistent current produced by Na_v_1.6 may play a role in a damaging injury cascade when coexpressed with Na^+^/Ca^+^ exchanger in demyelinated axons.	[193, 195, 197]
	Na_v_1.7	Gain-of-function mutation	Neuropathic pain	Patients	Hyperexcitability of neurons; acute or chronic pain.	[28, 195, 198, 199]
		Loss-of-function mutation	Congenital insensitivity to pain	Patients	Indifference to pain.	[28, 195, 198–200]
	Na_v_1.8	Gain-of-function mutation	Neuropathic pain	Patients	Mutations contribute to painful peripheral neuropathy by enhancement of the channel's response to depolarization and produce hyperexcitability in DRG neurons.	[193, 195, 201]
	Na_v_1.9	Gain-of-function mutation	Neuropathic pain	Patients	Gain-of-function mutations in this channel are suggested to contribute to pain, autonomic dysfunction, and axonal degeneration in patients with peripheral neuropathy.	[193, 195, 202]

Potassium channels	K_ir_6.1	Punctual mutation (SNP)	Coronary microvascular dysfunction and ischemic heart disease (IHD)	Patients	The polymorphism rs5219_AA of K_ir_6.2 is associated with a protective effect in the development of IHD.	[34, 193]
	K_Ca_1.1	Overactivation	Alzheimer disease (AD)	Mouse model	Increased availability of ROS in mouse models of AD, so BK channels are extensively oxidized.	[203]
	K_Ca_3.1	Overexpression	Diabetic nephropathy	Mouse model and human tissue	Knockout of K_Ca_3.1 reduces renal fibrosis in a mouse model of diabetic nephropathy.	[204]

Voltage-gated chloride channels (VGClCs)	CLIC1	Single nucleotide polymorphisms	Idiopathic epilepsy	Patients	Possible contribution of the “skeletal” chloride channel ClC-1 to the regulation of brain excitability.	[205]

Acid-sensing ion channels	ASIC1a	Overexpressed	HCC	Liver tumor tissues and SMMC-7721 cells	Suppression of ASIC1*α* expression by RNAi attenuates the malignant phenotype of HCC cells.	[206]

**Table 2:** Ion channels involved in oxidative stress in the liver.

Ion channel	Pathology	Model	Oxidative stress effect	Reference
K_v_2.1	Hepatoma	Huh-7 cell line	HCV inhibits K_v_2.1, suppressing apoptosis in response to oxidative stress.	[24]

K_ir_6.2	Acute liver injury	LPS-induced mouse model of liver injury	K_ir_6.2 knockout exacerbates LPS-induced endoplasmic reticulum stress in the liver.	[207]

TRPM2	Acetaminophen-induced liver damage	TRPM2 KO mice	H_2_O_2_^−^ and acetaminophen-activated Ca^2+^ entry is attenuated in TRPM2 KO mouse hepatocytes.	[208]

TRPM7	Liver fibrosis	Rat hepatic stellate cells	Blockage of TRPM7 causes HSC death induced by ER stress-mediated apoptosis.	[138]

TRPV4	Liver fibrosis	Human liver fibrotic tissues HSC-T6 cells Rat liver fibrosis model, CCl_4_	TRPV4 expression correlates with HSC activation and in HSC-T6 induction of *α*-SMA and Col1*α*1.	[144]

P2Y	Liver fibrosis	Rat liver fibrosis model, CCl_4_	Blockage of P2Y receptors inhibited CCl_4_-induced liver fibrosis in rats.	[131]

P2X7	Liver fibrosis NASH	Mouse liver fibrosis model, CCL_4_ Mouse diet-induced obesity (DIO)	P2X7 blockage attenuates mouse liver fibrosis and P2X7 gene-deleted mice decreased *α*-SMA, Col1*α*1, and TGF-*β*1 in DIO treated mice.	[117, 133]

CLIC1	Hepatocarcinoma	Mouse hepatocarcinoma ascites cell line (Hca-F)	Overexpression of CLIC1 contributes to cell proliferation, apoptosis, migration, and invasion.	[170]

ASIC1a	Liver fibrosis	Rat liver fibrosis model, CCl_4_	ASIC1a increases in HSC and inhibition of ASIC1a suppresses PDGF-induced profibrogenic effects of activated HSC.	[160]

VSOR	Hepatoma	Rat hepatoma (HTC) cells	Activated by H_2_O_2_ regulating cell volume and cell proliferation.	[170]

VDAC	Acute ethanol intoxication	Rat primary hepatocytes	Bax interacts with the PTP component protein VDAC and likely causes PTP opening, cytochrome c release, caspase activation, and apoptosis.	[209]

## Abbreviations

ASICs: Acid-sensing ion channels
ANT: Adenine nucleotide translocator
ADH: Alcohol dehydrogenase
ALD: Alcoholic liver diseases
ALDH: Aldehyde dehydrogenase
*α*-SMA: Alpha-smooth muscle actin
2-APB: 2-Aminoethoxydiphenyl borate
Bz-423: 1,4-Benzodiazepine
K_ATP_: ATP-sensitive potassium channels
K_Ca^2+^_: Calcium-activated potassium channels
CCl_4_: Carbon tetrachloride
CLIC1: Chloride intracellular channel 1
Col1*α*1: Collagen type 1 alpha 1
CsA: Cyclosporin A
DIDS: Diisothiocyanatostilbene-2,2′-disulfonic acid disodium salt hydrate
ER: Endoplasmic reticulum
EGF: Epidermal growth factor
ECM: Extracellular matrix
ERK1/2: Extracellular-signal-regulated kinases 1 and 2
P2Y: G protein-coupled purinergic receptors 2Y
HSCs: Hepatic stellate cells
HBV: Hepatitis B virus
HCV: Hepatitis C virus
HCC: Hepatocellular carcinoma
HTC: Hepatoma cells
HIV/AIDS: Human immunodeficiency virus infection and acquired immune deficiency syndrome
HER: Hydroxyethyl radical
H_2_O_2_: Hydrogen peroxide
^•^OH: Hydroxyl radical
HNE: Hydroxynonenal
HIF-1*α*: Hypoxia-inducible-factor-1*α*
IAA-94: Indanyloxyacetic acid
IMAC: Inner membrane anion channel
IMM: Inner mitochondrial membrane
IL-1*β*: Interleukin-1*β*
IK_Ca_: Intermediate-conductance Ca^2+^ activated K^+^ channel
JNK: c-Jun N-terminal kinases
KCs: Kupffer cells
K_ATP_: ATP-sensitive K^+^
P2X: Ligand-gated purinergic receptor
VOCC: L-type voltage-operated Ca^2+^ channels
MAPK: Mitogen-activated protein kinases
MDA: Malondialdehyde
MMPs: Matrix metalloproteinases
MCAO: Middle cerebral artery occlusion
MAMs: Mitochondria associated-endoplasmic reticulum membranes
mitoK_ATP_: Mitochondrial ATP-sensitive K^+^
MEOS: Microsomal ethanol oxidation system
PT: Permeability transition
MLK3: Mixed lineage kinase 3
NAFLD: Nonalcoholic fatty liver disease
NASH: Nonalcoholic steatohepatitis
NADH: Nicotinamide adenine dinucleotide (reduced)
NO: Nitric oxide
NPPB: 5-Nitro-2-(3-phenylpropyl-amino)benzoic acid
NF-kappa-B: Nuclear factor kappa-light-chain-enhancer of activated B cells
OMM: Outer mitochondrial membrane
PTP: Permeability transition pore
ONOO^−•^: Peroxynitrite
PLC*γ*1: Phospholipase C gamma 1
PDGF-BB: Platelet-derived growth factor
PcTX1: Psalmotoxin
PPADS: Pyridoxal-phosphate-6-azophenyl-2′,4′-disulfonate
ROS: Reactive oxygen species
RNS: Reactive nitrogen species
GSH: Reduced glutathione
RVD: Regulatory volume decrease
Rh123: Rhodamine 123
SECs: Sinusoidal endothelial cells
SK_Ca_: Small-conductance Ca^2+^ activated K^+^ channel
O_2_^−•^: Superoxide anion
TIMPs: Tissue inhibitors of metalloproteinases
TGF-*β*1: Transforming growth factor-beta-1
TRP: Transient receptor potential
TRPM7: Transient receptor potential melastatin-like 7
TRPV4: Transient receptor potential vanilloid 4
TNF-*α*: Tumor necrosis factor alpha
TPCs: Two-pore channels
VDAC: Voltage dependent anion channel
VSOR: Volume-sensitive outwardly rectifying.
